# Real-time Acute Stress Facilitates Allocentric Spatial Processing in a Virtual Fire Disaster

**DOI:** 10.1038/s41598-017-14910-y

**Published:** 2017-11-03

**Authors:** Zhengcao Cao, Yamin Wang, Liang Zhang

**Affiliations:** 1 Beijing Key Laboratory of Learning and Cognition, Department of Psychology, Capital Normal University, Beijing, 100037 China; 2 State Key Laboratory of Brain and Cognitive Science, Institute of Psychology, Chinese Academy of Sciences, Beijing, 100101 China; 3 University of Chinese Academy of Sciences, Beijing, 100049 China

## Abstract

Prior studies have shown that spatial cognition is influenced by stress prior to task. The current study investigated the effects of real-time acute stress on allocentric and egocentric spatial processing. A virtual reality-based spatial reference rule learning (SRRL) task was designed in which participants were instructed to make a location selection by walking to one of three poles situated around a tower. A selection was reinforced by either an egocentric spatial reference rule (leftmost or rightmost pole relative to participant) or an allocentric spatial reference rule (nearest or farthest pole relative to the tower). In Experiment 1, 32 participants (16 males, 16 females; aged from 18 to 27) performed a SRRL task in a normal virtual reality environment (VRE). The hit rates and rule acquisition revealed no difference between allocentric and egocentric spatial reference rule learning. In Experiment 2, 66 participants (32 males, 34 females; aged from 19 to 30) performed the SRRL task in both a low-stress VRE (a mini virtual arena) and a high-stress VRE (mini virtual arena with a fire disaster). Allocentric references facilitated learning in the high-stressful VRE. The results suggested that acute stress facilitate allocentric spatial processing.

## Introduction

Since the concept of cognitive mapping was introduced by Tolman in 1948^1^, the nature of spatial learning has been a long-standing debate within the spatial cognition literature, and this debate has now become the core theoretical topic of issue in current spatial cognition and cognitive neuroscience^2–5^. One theory views spatial learning as incidental learning (also known as cognitive map learning), while another theory holds that spatial learning is a type of stimulus-response learning (also known as associative learning)^4^. Correspondingly, there are two central theories on the representations of spatial relationships. One framework, labeled as proximal and distal referencing, is a stimulus-associated frame of reference and codes space by distance from the associated stimuli^6–9^. The other framework views spatial relationships as either relative to the body (egocentric) or relative to an object (allocentric)^1,10–19^. An allocentric frame of reference functions by specifying location and orientation independent of body position and as such, spatial representations are centered on objects or environmental features compared to self-centered egocentric frame of reference^17,20,21^.

The effects of stress on spatial learning provides fundamental evidence for the core theoretical question from an evolutionary perspective. Adaptive response to acute stress is of prime importance to immediate survival for both humans and animals. Spatial learning strategy, the ability that allows humans or animals to efficiently locate food, partners or avoid dangers as soon as possible, has been linked to the adaptation to environmental stress^22–26^. Studies in both animals and humans, have revealed that exposure to stress *prior to* a task affects spatial learning in a virtual or real Morris water maze^6–9^. For example, Schwabe *et al*.^27–29^ examined the effect of stress on two learning strategies in spatial navigation: stimulus-response strategy and spatial learning strategy. Stimulus-response learning is a kind of associative learning while spatial learning used by them belongs to incidental learning. They demonstrated that stress facilitates spatial navigation learning by stimulus-response learning strategy (proximal reference) but not by spatial learning strategy (Distal reference, incidental learning). But, other studies have demonstrated that exposure to stress prior to a task impairs proximal spatial processing^30–32^, and still others have found no influence of stress on proximal or distal spatial processing^33,34^. For example, using a virtual reality maze, Guenzel, *et al*.^31^ demonstrated that exposure to stress prior to the task impaired, rather than facilitated stimulus-response learning (proximal reference).

To our knowledge, to date, no study has examined the effects of stress on egocentric and allocentric spatial processing. Nevertheless, a frequently encountered problem in human life is how to find a way out. Some people get used to coding spatial relations relative to himself (egocentric, right or left to oneself) while others like using a landmark (allocentric: far or near to a land mark). Then, during times of acute stress, such as a fire disaster, whether egocentric or allocentric processing presides remains unknown. From an evolutionary point of view, a specific mechanism to cope with acute stress would be expected.

Particularly, prior studies investigating the effect of stress on spatial learning have most frequently presented the stressor prior to the cognitive task, while far fewer studies have presented the stress in real-time^6–9,35^. Learning that takes place after a stressful situation is very different from learning that occurs during a stressful situation. Acute stress triggers a series of complex neuroendocrine responses. The stress response consists of both physiological (neuroendocrine) and psychological (emotional and cognitive)^36,37^ components. In addition, the stress response involves both the fast acting sympathetic nervous system and the slower hypothalamus-pituitary-adrenal (HPA) axis^38^. During acute stress, the activation of the two systems occurs quickly and then rapidly returns to baseline. For example, the heart rate, which indexes the sympathetic nervous system, declines to baseline immediately following acute stress^39^. Therefore, the effects of acute stress on cognitive functioning are very different depending on when the stress is experienced (during stress or after the stress). The exploration of cognitive functioning during real-time stress can provide new data to what is already known about stress and spatial learning. Recently developed immersive virtual reality technique helps to create a vivid VR-based stress VRE. Immersive virtual reality technique contains a real-time tracking system and a panoramic system, which can provide a controllable real-time stressful environment with immersive user experience. Thus, it is possible now to observe participants’ response in a real-time natural stress.

Cognitive neuroscience research has revealed that stress affects proximal/distal spatial processing via the caudate nucleus and the hippocampus. The caudate nucleus is involved in processing the less cognitive “stimulus-response” or “habit” type of learning, while the hippocampus primarily mediates more cognitive spatial learning^2–4,16,40–42^. In addition, other research offers a dual representation theory, in which the striatal system is recruited in egocentric, involuntary and depictive learning, while the hippocampus primarily mediates incidental learning which is considered to be allocentric, voluntary and abstract^2–4,16,40–42^. There is therefore a consensus that the striatal system is used in stimulus-response learning while the hippocampus processes incidental learning.

There is some confusion in terminology within the field. According to the definition of the allocentric reference frame, proximal and distal referencing both belong to the allocentric frame of reference. The question therefore arises, if striatum-based learning is egocentric^2–4,16,40–42^, then, do the findings that stress facilitates proximal spatial processing^27–29^ predict facilitation of allocentric spatial processing or egocentric spatial processing?

The purpose of the present study was to investigate the effects of acute real-time stress on egocentric and allocentric spatial processing. An immersive virtual reality-based fire disaster was created to simulate a natural acutely stressful condition. The immersive virtual reality technique has been recently developed to provide real-time tracking in a vivid simulated environment, which provide ultimate level of immersion of real or imagined environments. Here, we simulate acute stress along with a reward-based spatial learning task. In the present study, a virtual disaster was designed that included a large fire, a flood and falling bricks, during which participants were instructed to perform an SRRL task. There were four blocks of trials consisting of one rule each. During the experiment, participants were instructed to make a location selection by walking to one of three poles. The participant’s selection was rewarded when the selected pole was in accordance with the assigned rule within the block. Two egocentric and two allocentric reference rules were assigned. The rate of correct responses (hit rate) and rule acquisition (RA) in egocentric versus allocentric reference rule blocks were compared. The effect of stress was examined by comparing differences in learning efficiency between the high-stress and low-stress virtual reality environments (VREs).

## Results

### Assessment of VREs and stress

The mean Igroup Presence Questionnaire (IPQ) score in all four dimensions (Presence, Spatial Presence, Involvement and Experienced Realism) of the normal VRE was above 2.5 and in the stressful VREs was above 3, indicating a valid user experience in the normal and stressful VREs. These results are comparable to those of other existing VR games^43,44^.

There was a significant difference (t = −8.441, df = 63, *p* < 0.001) in the self-reported stress assessment between the high-stress environment (mean score 3.578) and the low-stress environment (mean score 2.203). The reported stress in the high-stress environment was also significantly different from that reported in the normal environment (mean score 1.438, t = −9.709, df = 94, *p* < 0.001). These results indicate the validity of the stressful experience.

### Experiment 1 Rule learning in normal VRE

A paired t-test of reference type (egocentric and allocentric) on hit rate and RA showed no differences between the two reference types in hit rate or RA (hit rate: t = 0.478, df = 31, *p* = 0.636; RA: t = 0.530, df = 31, *p* = 0.600), indicating that learning in the normal environment did not differ between egocentric and allocentric references (Table 1).

**Table 1:** Performance in SRRL task in normal VRE.

	**Egocentric**	**Allocentric**
Hit Rates	0.64 (0.04)	0.61 (0.04)
RAs	0.52 (0.08)	0.44 (0.08)
Data shown as Mean (SEM)		

Learning among the four reference rules was further examined by a repeated measures ANOVA of reference rules (leftmost, rightmost, nearest and farthest) on hit rates and RAs. There were no differences among the four reference rules (hit rate: F _(3, 29)_ = 0.287, *p* = 0.835, η2 p = 0.029; RAs: F _(3, 29)_ = 0.845, *p* = 0.481, η2 p = 0.080), indicating no rule preference.

### Experiment 2 Rule learning in stressful VREs

#### Hit Rates

A 2 (Stress Level: low, high) × 2 (Reference Type: egocentric, allocentric) ANOVA showed no main effect of stress level or reference type on hit rate. However, there was a significant interaction between stress level and reference type (F _(1, 62)_ = 6.404, *p* = 0.014, ${{\rm{\eta }}}_{{\rm{p}}}^{2}$ = 0.092). A simple effects analysis indicated that the hit rates were significantly lower when egocentric references were used than when allocentric references were used in the high-stress condition (High-Stress: Egocentric = 0.578, Allocentric = 0.694, F _high stress (1,63) = _5.710, *p* = 0.020, ${{\rm{\eta }}}_{{\rm{p}}}^{2}$ = 0.091; Low-Stress: Egocentric = 0.601, Allocentric = 0.589, F _low stress (1,63) = _0.070, *p* = 0.794, ${{\rm{\eta }}}_{{\rm{p}}}^{2}$ = 0.002). In addition, the hit rates in the allocentric reference rule learning condition in the low-stress condition were lower than those in the high-stress condition (F _allocentric (1,63)_ = 8.540, *p* = 0.005, ${{\rm{\eta }}}_{{\rm{p}}}^{2}$ = 0.135; F _egocentric (1,63)_ = 0.410, *p* = 0.522, ${{\rm{\eta }}}_{{\rm{p}}}^{2}$ = 0.007) (Table 2). These results indicate that a high stress level selectively facilitates allocentric reference rule learning.

**Table 2:** Performance in SRRL task in stressful VREs.

	**Low Stressful VRE**		**High Stressful VRE**	
	**Egocentric**	**Allocentric**	**Egocentric**	**Allocentric**
Hit Rates	0.60 (0.03)	0.59 (0.03)	0.58 (0.03)	0.69 (0.03)
RAs	0.50 (0.06)	0.48 (0.06)	0.41 (0.06)	0.69 (0.06)
Data shown as Mean (SEM)				

#### RAs

A 2 (Stress Level: low, high) × 2 (Reference Type: egocentric, allocentric) ANOVA showed no main effect of stress level or reference type on RA. However, there was a significant interaction between stress level and reference type on RA (F _(1, 62)_ = 8.196, *p* = 0.006, ${{\rm{\eta }}}_{{\rm{p}}}^{2}$ = 0.115). A simple effects analysis found that the RAs under the egocentric rule learning condition were lower than those under the allocentric rule learning condition in the high-stress situation (Egocentric RA _high stress_ = 0.406, Allocentric RA _high stress_ = 0.688, F _high stress (1,63)_ = 7.790, *p* = 0.007, ${{\rm{\eta }}}_{{\rm{p}}}^{2}$ = 0.124; Egocentric RA _low stress_ = 0.500, Allocentric RA _low stress_ = 0.484, F _low stress (1,63) = _0.020, *p* = 0.877, ${{\rm{\eta }}}_{{\rm{p}}}^{2}$ < 0.001). In addition, the RAs during the allocentric rule learning condition in the low-stress environment were lower than those in the high-stress environment (F _allocentric 1,63_ = 9.060, *p* = 0.004, ${{\rm{\eta }}}_{{\rm{p}}}^{2}$ = 0.144; F _egocentric 1,63_ = 1.650, *p* = 0.203, ${{\rm{\eta }}}_{{\rm{p}}}^{2}$ = 0.026) (Table 2).

#### Results of trial by trial analysis in normal and stressful VREs

Prior evidence suggests that errors can have effects within and beyond the time course of a trial^45^. To further investigate the possibility of inter-trial effects of errors in the rule learning task, we split the participant responses into four groups based on the connection between the feedbacks from one trial to the other (previous trial to present trial). The four groups were: wrong previous-trial and wrong present-trial (WW), wrong previous-trial and right present-trial (WR), right previous-trial and wrong present-trial (RW), and right previous-trial and right present-trial (RR).

In the normal and low-stress VREs, trial-by-trial analysis of consecutive responses in each experimental condition revealed that the response of the present trial was not significantly affected by the feedback from the previous trial in all conditions (leftmost rule, rightmost rule, nearest rule, and farthest rule learning). However, in the high-stress VRE, the responses in the present trial were significantly affected by the feedback from the previous trial during allocentric learning (χ^2^_(1)_ = 4.296, *p* = 0.038) and there was a tendency toward this effect during egocentric learning (χ^2^_(1)_ = 3.556, *p* = 0.059).

## Discussion

The present study investigated whether acute stress affects allocentric and egocentric spatial processing in a simulated stressful environment. The results show that hit rates and RAs were significantly greater when allocentric reference rule learning was used in high-stress, rather than normal or low-stress VREs. No differences were found between egocentric and allocentric reference rule learning in other conditions. The trial by trial analysis also confirmed a significant inter-trial effect in the high-stress VRE. The mean hit rates in all conditions were above probability level, indicating that rule learning occurred. The present study is the first to show a difference in performance under egocentric and allocentric spatial processing in a simulated natural disaster. In addition, several other important issues in the field of spatial learning research are discussed.

Considering the long-standing debate on the nature of spatial learning, the current finding that acute stress facilitates allocentric but not egocentric spatial reference processing is in agreement with previous studies^27–29,46^. Existing studies have investigated whether stress prior to task affects stimulus-response learning (proximal spatial reference or intra-maze feature reference) and incidental learning (distal spatial reference or environmental boundary feature reference), and have revealed that stress facilitates stimulus-response learning^5,8,9,28,30,47^. For example, Schwabe *et al*.^27–29^ demonstrated that stress facilitates spatial navigation learning by stimulus-response learning strategy (proximal reference) but not by incidental learning strategy (distal reference). However, in the present study, allocentric spatial references were made relative to the central tower in the arena and the closest distance from the participant to the tower was less than 1.5 meters. Thus, allocentric referencing in the current study is essentially the same as proximal intra-maze referencing used in previous studies. In this sense, our results are inline with previous findings. The current study therefore, places the terminological confusion at the forefront of the field. The confusion may lead to a misunderstanding of the stress effect on spatial learning, especially when neural substrates are considered. For example, prior studies have linked proximal spatial processing (or far/near reference) to striatum-based learning^5,27–29^, leading to difficulty in associating striatal learning with egocentric spatial learning. The separation of egocentric and allocentric spatial processing has been supported by increasing evidence from both neuropsychological and cognitive neuroscience research^4,5,48–51^, and as such, it is unreasonable to associate both egocentric and allocentric spatial processing to the striatal system. From an evolutionary perspective, the present study supports a potential mechanism for egocentric referencing that may not be constrained to the body of the viewer. In cognition, both egocentric and proximal reference could be viewed as associative response of stimulus-response learning regardless of the debate of stimulus-response learning versus incidental learning. They are both used to encode the proximal space of a stimulus. Thus, using associative spatial reference instead of egocentric and proximal reference might resolve the conflicts. In the future, further research might be designed to explore the possible difference between proximal and egocentric reference in spatial learning.

Our findings are also in agreement with prior neuroscience research. A large number of cognitive neuroscience studies have revealed that either proximal/distal or egocentric/allocentric spatial reference frames are involved in two neural modules; the striatal system and the hippocampal system, irrespective of potential complexity^2–4,16,40–42,52^. Based on these studies, the striatal system is recruited during stimulus-response learning which has also been described as egocentric, involuntary, depictive and less cognitive learning. On the other hand, the hippocampus primarily mediates incidental learning which has been described as allocentric, voluntary, abstract and more cognitive learning. Functionally, the striatum serves to automatically associate immediate actions to specific stimuli or landmarks that predict reward, while the hippocampus links objects to environmental locations where the objects are encountered.

Previous studies have suggested that stress, or a high corticosterone (cortisol in human) level resulting from stress, impairs hippocampal functioning, while enhancing striatal functioning. For example, stress impairs incidental learning (hippocampal learning) and facilitates stimulus-response learning (striatal learning)^27–29,46,47^. In the current study, allocentric referencing was made relative to a central tower, which is similar to proximal referencing used in previous studies^27–29,46^. Thus, the effect of stress in the present study can be interpreted as striatal enhancement. A fire disaster, as a real-time stressor, would immediately activate amygdala functioning which would then integrate cognitive processing associated with emotionally salient stimuli. These steps would lead to an involuntarily attention to salient environmental events and result in the facilitation of allocentric spatial processing. However, terminological confusion has emerged among existing studies. Prior researchers who have investigated proximal and distal reference frames, have proposed that the striatal system is egocentric. However, the definition they use is actually more similar to allocentric referencing used in the current study^2^. Given that allocentric referencing was defined as an object-centered reference frame, proximal and distal reference frames are both object-centered and therefore allocentric. The confusion in terminology is derived from the long-standing core debate about the nature of spatial learning (as discussed in the introduction). To solve confusion within the field, we propose that the complexity of the connection between the striatal and hippocampal systems^4^ and the potential function of the hippocampus^53^ may provide a new direction for elucidation.

Prior studies have shown that humans represent spatial relations egocentrically rather than allocentrically in a normal environment^40,54,55^. For example, in a probabilistic learning task, researchers examined whether participants were more likely to use a viewer-centered reference frame or an environment-centered reference frame^54^. The results showed that participants preferentially rely on egocentric rather than allocentric spatial processing. Based on the current work, we argue that inconsistencies might be derived from the use of table-based tasks. In a table-based task, it is necessary for participants to focus their attention on the table rather than the surroundings, which thus leads to an egocentric reference. Notably, table-based tasks have been widely used in exsiting studies conducted in both normal and stressful situations^28,40,54,55^.

Real-time stress induction was confirmed by high scores on the stress scale. In the current experiment, stress scale scores were greatest after the high-stress condition. In addition, there was a significant positive correlation between stress scores of the low- and high-stress environments (r = 0.277, p = 0.027). In addition, self-reported emotional distress were high in the high-stress condition, and were consistent with previous studies^56,57^. The detailed data show that all three events in the current study were stressful (big fire: 2.719 on a five-point Likert scale, flood; 2.53, and falling bricks: 2.344).

An interesting question has arisen from the present study is how people find a way out in a fire disaster. The findings of present study support that human participants will code spatial relations by referencing landmark (far or near to an object) rather than one’s body (right or left to oneself). The results can be interpreted by a recently developed dual-network theory (salience network versus executive control network)^58^. Hermans, *et al*.^58^ proposed that exposure to acute stress lead to a reallocation of resources to a salience network, which integrates cognitive processing associated with salient stimuli, including exogenous attention. In a fire disaster, the salience network thus lead to object-based processing for immediate survival. After stress subsides, as Hermans, *et al*.^58^ proposed, resource reallocate to executive control network to enhance higher order cognitive processes important for long-term survival. Thus it can be seen how our cognition adapts to stressful environment.

While the current study provides novel and interesting data to the field of spatial learning, there are limitations to the study. The first limitation is that distal references were not considered in our design. In future research, it will be necessary to improve the experimental design to include comparisons between proximal and distal references. Second, strict stimulus control was not possible within the ecologically valid design. A method to reduce physical salience of stressors while maintaining emotional salience is necessary for future research.

In Summary, the present study aimed to enhance the ecological validity of laboratory experiments investigating the effects of stress on spatial learning. From an evolutionary perspective, we observed participant spatial strategy during a simulated natural disaster. In line with previous findings, allocentric learning strategy (proximal reference) facilitated learning in a stressful environment. Our study is the first case that simulated natural disaster and observed how the real-time stress affect spatial learning strategy. Although cortisol reach its high level within 30 minutes after the onset of stress, it has practical value to observe response under acute stress. Although a causal relationship cannot be determined, the real-time acute stress closely simulated a natural setting and ecological validity was therefore achieved.

## Methods

All experimental procedures were approved by the Capital Normal University Institutional Review Board. Methods were carried out in accordance with relevant guidelines and regulations. All participants signed an informed consent approved by the Capital Normal University Institutional Review Board. All participants were monetarily compensated.

### Participants

A gender balanced sample of 98 healthy participants with normal or corrected-to-normal vision were recruited from the Capital Normal University and other universities. Eligible participants indicated via a questionnaire that they did not have aquaphobia or pyrophobia, and that they had experience with 3D games. Thirty-two participants (16 males, 16 females; aged from 18 to 27) completed Experiment 1(normal, non-stressful VRE), and 66 (32 males, 34 females; aged from 19 to 30) participants were enrolled in Experiment 2 (stressful VRE). Two female participants were excluded from Experiment 2 due to illness. To maintain a consistent level of stress-related hormones, all participants were tested in the afternoon between the hours of 1:00 and 4:30 pm^59^.

### Apparatus

The experiment was performed with a virtual reality system consisting of a head-mounted virtual reality helmet (Oculus Rift DK2, Oculus, Irvine, CA, U. S.; 100° horizontal field of view, resolution 960 × 1080 pixels per eye, refresh rate 75 Hz; delay 2~3 ms), an HP workstation (CPU E5–2667 3.20 GHz, RAM 56.0 GB, GPU NVIDIA Quadro K6000), and an infrared tracking subsystem. The infrared tracking subsystem was comprised of eight intelligent tracking cameras (ART Track 5 tracking cameras; refresh rate 300 Hz) and one central controller with DTrack2 software (ART technology company, Weilheim, Germany). The tracking cameras covered a 6 m × 6 m tracking area on the floor. The virtual reality environment (VRE) was presented on the head-mounted displays, which was tracked by the infrared tracking subsystem via a pair of glasses fixed with six passive markers. The sounds in the experiment were presented by a stereo subsystem (Wharfedale Pacific Evolution-40, Wharfedale, Britain).

### Stimuli

**Figure 1:**
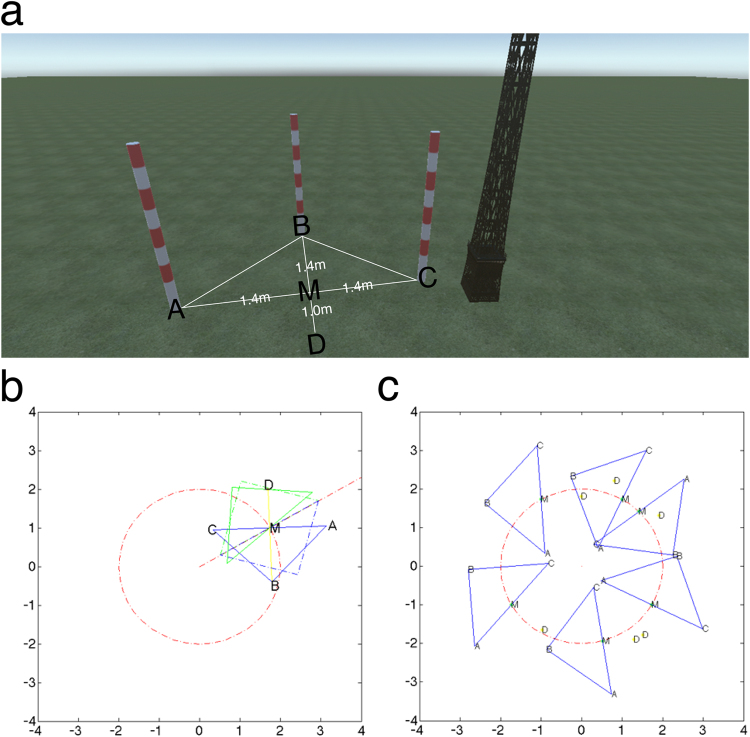
Depiction of SRRL task. (**a**) Triangle route in virtual reality environment with point “D” as the starting point of each trial. The tower was always presented in the center of the scene. (**b**) Circular area and triangular route with the “M” point as the midpoint of the hypotenuse of the triangles. (**c**) Example of six triangular routes; three clockwise and three counterclockwise. See Supplementary video S1 for the demonstration of normal VRE.

Stimuli consisted of three virtual red poles, one virtual green pole, an iron tower, and two stressors. The red poles and the green pole were identical in shape and size (10 cm radius, 1.8 m tall). The green pole was used as a starting point while the three red poles were used as selection poles (see Fig. 1a). The iron tower (7.34 m tall, width 33 cm) was placed at the center of the virtual field and served as a landmark. Several sounds were used as feedback stimuli in both Experiment 1 and Experiment 2. The stressors used in present study were a low stressful VRE of an arena with night sky, and a high stressful VRE in which a virtual fire disaster was added to an arena.

### Triangle routes

To inhibit the influence of location and direction selection in the VRE, thirty-six triangular routes with the same shape and size were selected around the tower (see Fig. 1). Triangular routes were selected via a three-step procedure. First, a circle (2 m radius) was created with the tower at the center. Second, six points (e.g. see “M” in Fig. 1a), equally distanced around the perimeter of the circle (randomized between 1-m to 2-m), were selected (see Fig. 1c). Third, at each “M” point, a clockwise and counterclockwise triangle was created with the “M” as the midpoint of the hypotenuse of the triangle. Finally, each of the triangles was rotated either clockwise or counterclockwise at a random angle between −15° and 15° around “M” to create a new triangular route, (see Fig. 1b). Therefore, a total of 36 triangular routes were created as judgement poles. The 36 triangular routes were randomly divided into six groups with each group containing six routes, (for an example, see Fig. 1c). During the experiment, a selection pole appeared at each vertex of the triangular route while the starting pole at point “D” disappeared (see Fig. 1a).

### VREs

Two types of VREs (normal and stressful) were designed and created using Unity game development software (Unity Technologies, Inc., San Francisco, United States). A normal (no-stress) VRE was created for Experiment 1 and consisted of a blue skybox and an endless grass-textured ground. An iron tower was set at the center of the VRE serving as a landmark. The VRE was experienced as a 100° field of view. Navigation was controlled via a real-time tracking system.

**Figure 2:**
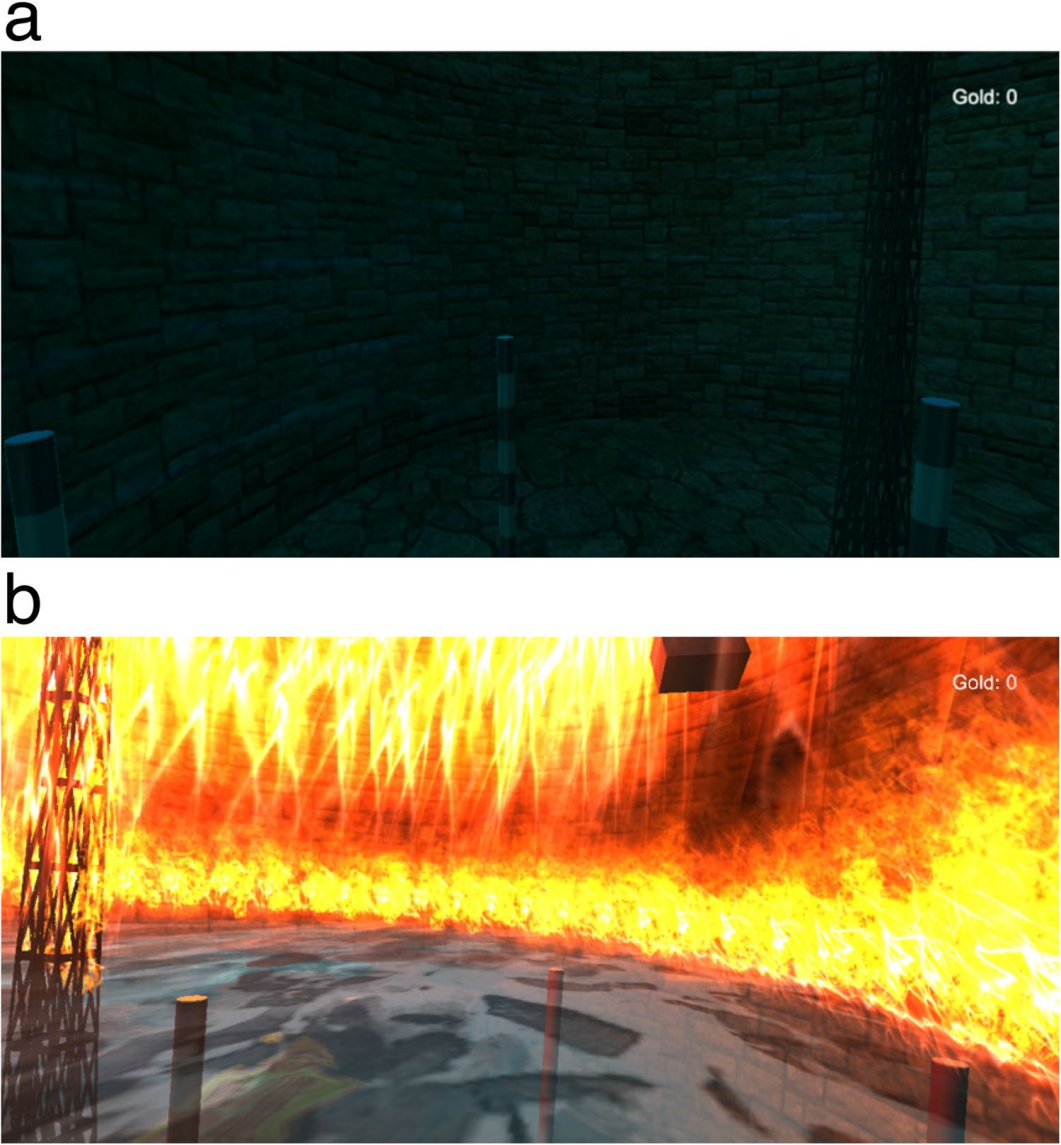
Screenshots of the low- and high-stress VREs. (**a**) low-stress VRE, similar to the normal VRE except for a stone ground, brick wall and night sky. (**b**) high-stress VRE, similar to low-stress VRE except for a flood, fire and falling bricks. See Supplementary videos S2 and S3 for the demonstration of low-stress (S2) and high-stress (S3) VREs.

The stressful VREs in Experiment 2 were divided into a low-stress VRE and a high-stress VRE. The low-stress VRE was created based on the normal VRE as described in Experiment 1. In the low-stress VRE, the blue sky of the normal VRE was replaced with a night-time sky, and a circular area (4.0 m in diameter) with a rock pattern was created on the ground around the tower. In addition, there was a brick wall around the area (6.40 m height) (see Fig. 2a). The high-stress VRE was identical to that of the low-stress VRE except that there was a fire disaster, including a large fire, a flood and falling bricks. The fire in the high-stress VRE consisted of a small fire burning on the top of the wall, and a large fire erupting 1.7 meters from the ground on the inside of the wall and the outside of the tower (1.7 meters from the ground). In addition, a large flood emerged and rose to 1.65 meters. The falling bricks occurred at randomized intervals between 3 s and 10 s during the fire disaster. The sounds of burning gas, falling rocks, flooding water, and earthquake rumblings were used to enhance the VRE effects.

### SRRL task

**Figure 3:**
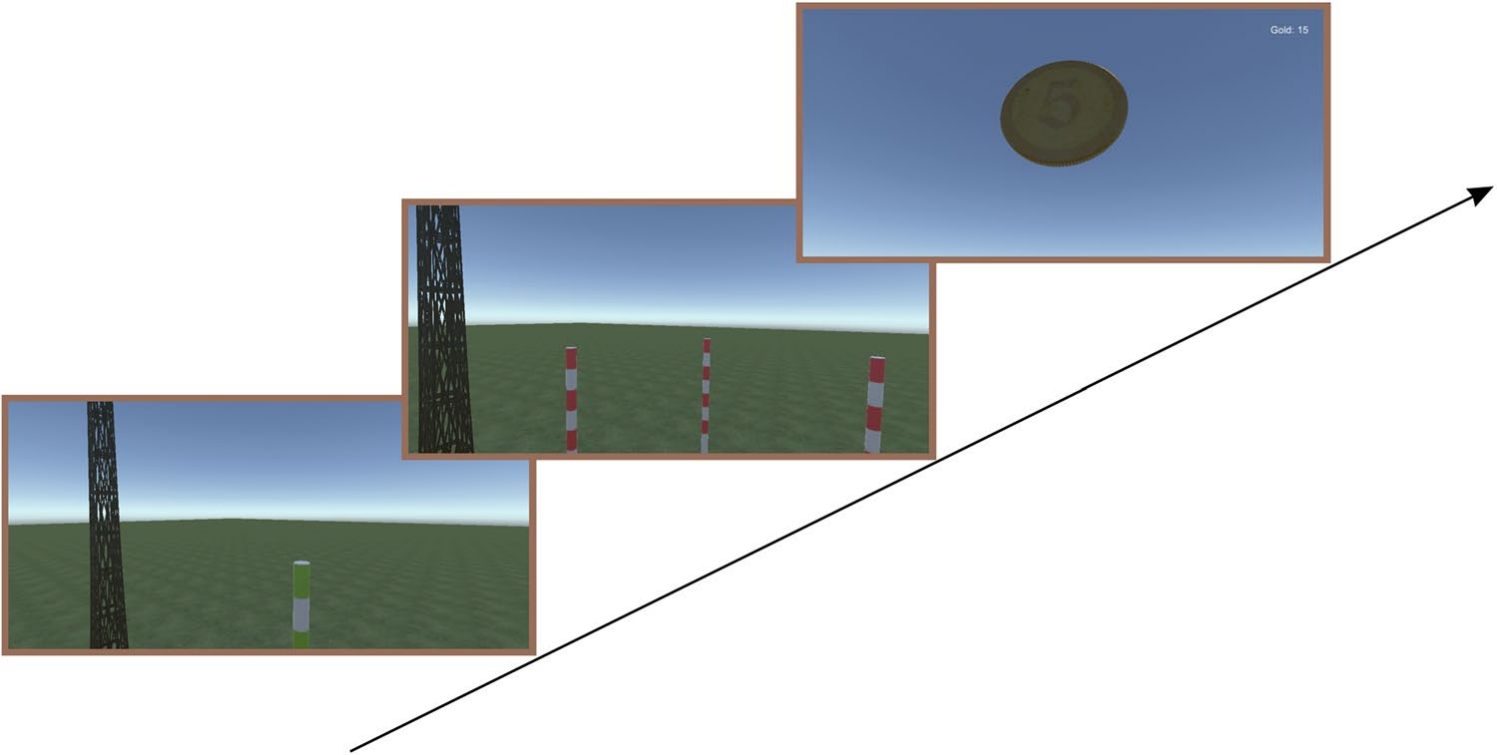
The SRRL task procedure. At first, a green pole appeared in the VRE and participants were instructed to walk to it. When the participant reached the green pole, three red selection poles appeared accompanied by the sound of a “bubble”. Participants were instructed to turn around to face the three red poles, and to choose one pole to walk to. Correct choice was immediately rewarded by a gold coin.

The SRRL task was the same in Experiments 1 and 2. Participants were motivated to learn spatial reference rules without any cues. The task began when a green pole appeared in the VRE and participants were instructed to walk to it. When the participant reached the green pole, three red selection poles appeared (as described above) accompanied by the sound of a “bubble”. Participants were instructed to turn around to face the three red poles, and to choose one pole to walk to. When the participant reached the selected pole, there was either a gold coin rotating over the pole accompanied by a “ringing” sound indicating a “correct” selection or the sound of a “toot” appeared, indicating a “wrong” selection. Each “correct” selection was rewarded with 0.07 RMB per coin after the experiment. Throughout the task, a small transparent panel at the top right corner of DK2 view displayed the total gold coins obtained in the block (Fig. 3). Two allocentric reference rules (leftmost reference and rightmost reference) and two egocentric reference rules (nearest and farthest reference) were used in the learning task.

### General design

Experiment 1 had a single factor (reference rule) design. Four specific reference rules (leftmost, rightmost, nearest, and farthest) were randomly arranged into four blocks, and the order of the blocks was counter-balanced for each participant. Each block contained 36 trials which were pseudo-randomly matched with the 36 triangular routes created prior to the experiment (described above). Thus, each participant completed a total of 144 trials (36 trials × 4 blocks) within 40 minutes. Between blocks, participants rested for two minutes.

Experiment 2 had a two-factor (stress level and reference type) within-subject design. As such, there were four conditions (low-stress-egocentric, low-stress-allocentric, high-stress-egocentric, and high-stress-allocentric) within each block of 36 trials. The order of the blocks was randomized for each participant. To avoid virtual reality sickness^60,61^ and habituation to stress^62^, participants in Experiment 2 remained in the stressful environment for no more than one hour. We therefore reduced the reference rules from two (e.g. rightmost- and leftmost-reference rules) to one (e.g. either rightmost-reference rule or leftmost-reference rule) in each of four conditions. The selections were counterbalanced among participants. Therefore, in Experiment 2, each participant learned an allocentric rule and an egocentric rule in both low- and high-stress VREs.

### Procedure

**Figure 4:**
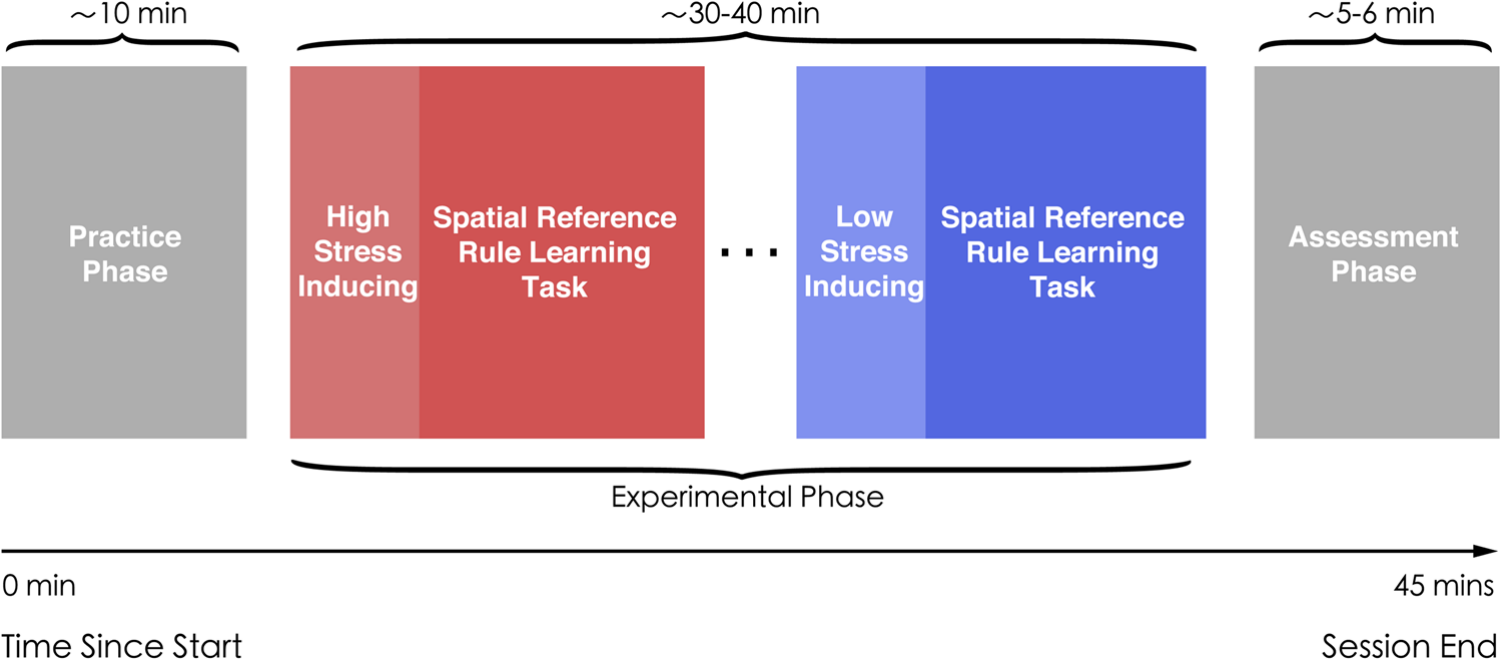
Learning procedure in Experiment 2. The red square and blue square illustrate examples of two blocks during the experimental phase. The red square depicts the high-stress environment while the blue square depict the low-stress environment. The order of the blocks was randomly assigned.

Both Experiment 1 and Experiment 2 contained three phases: a practice phase, an experimental phase and an assessment phase, see Fig. 4. During the practice phase, participants read instructions on the experimental task. An experimenter then helped with attaching the VR head gear in order to become familiar with the VRE. Participants then performed two practice trials which were identical to experimental trials of normal or stressful VRE. Immediately after the two practice trials, participants entered the experimental phase (as described above).

The trials in Experiment 2 differed from those in Experiment 1 at two points. First, in Experiment 2, the judgment poles disappeared after the participant’s selection or after 10 seconds in both the low- and high-stress conditions. In Experiment 1, the poles disappeared only after the participant’s selection. Second, the fire, flood and brick attacks emerged with the appearance of the red poles and disappeared after a correct selection only in the high-stress situation. There was no fire disaster in Experiment 1 or in the low-stress condition of Experiment 2.

### Assessment phase

After the experimental phase, participants completed a self-reported stress questionnaire. The stress scale^56,63^ contained one question (“I felt a kind of tension, fear or distress in the virtual circular arena”) on a five-point Likert scale to measure the stress induced by the VRE. After completing the stress questionnaire, participants completed the Igroup Presence Questionnaire (IPQ)^43,44^ to assess the feeling of presence experienced in the VRE. The Igroup Presence Questionnaire (IPQ) is a scale for measuring the sense of presence experienced in a virtual environment.

### Data analysis

**Figure 5:**
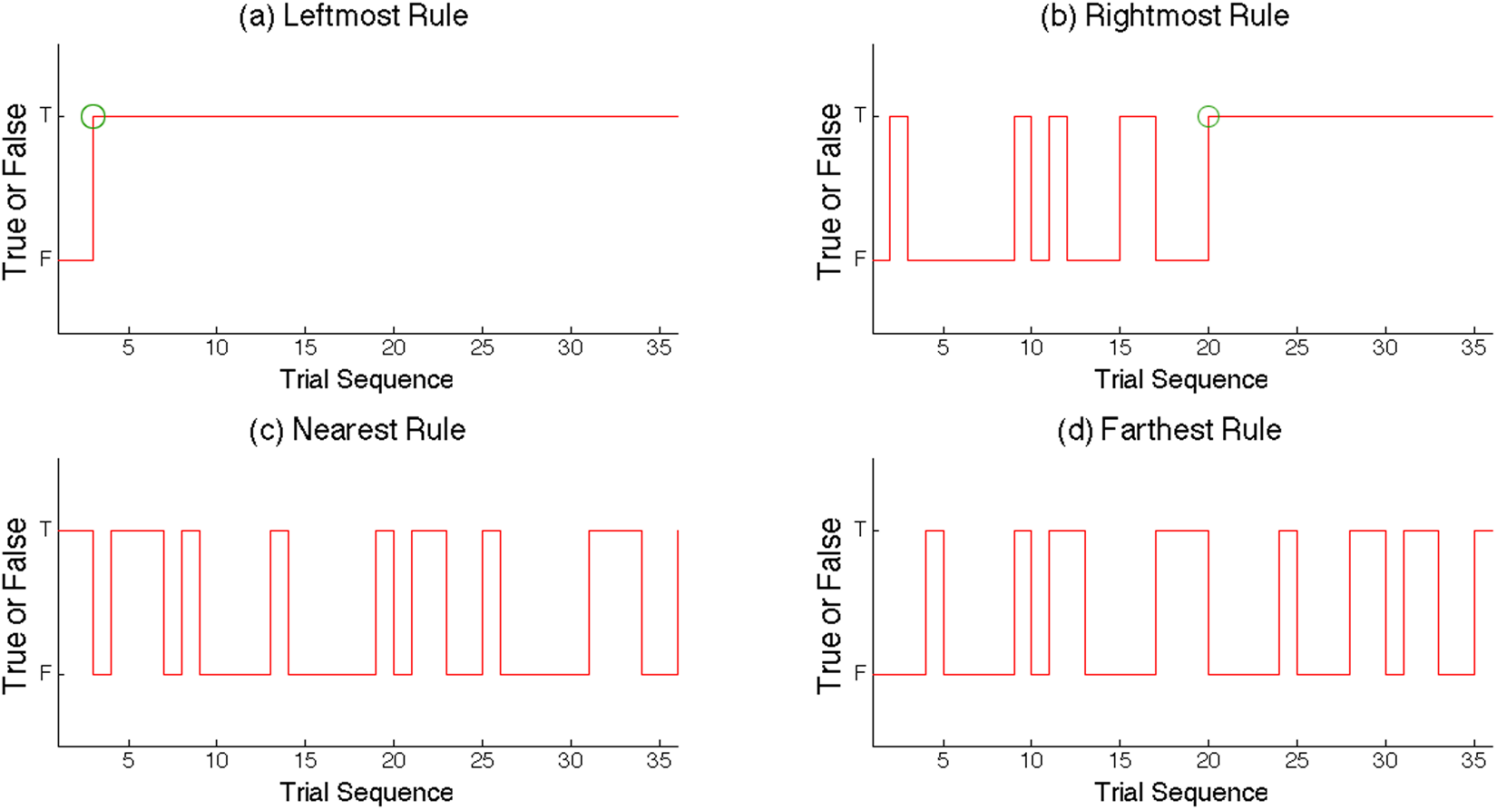
A sample of the RA algorithm of one participant. The green circles in (**a**) and (**b**) show two turning points detected by an algorithm. Turning point is used as an index of RA.

Hit rates and RAs were computed for analysis in the two experiments. The mean hit rate and RA for each participant were calculated for each block. Next, the mean RA for egocentric and allocentric rule learning were computed separately across the four blocks for each participant. A detection algorithm was used to judge whether participants had learned the rule assigned in a block. As prior study did, a turning point was detected by scoring rules^28^ (see Fig. 5). RA within a block was recorded as “1” when a participant made correct choices in six consecutive trials without changing in the following trials. When RA was not reached, a “0” was recorded.

## Electronic supplementary material

Supplementary Information

Supplementary Video S1

Supplementary Video S2

Supplementary Video S3
