# Publisher Correction: Real-time Acute Stress Facilitates Allocentric Spatial Processing in a Virtual Fire Disaster

**DOI:** 10.1038/s41598-018-20354-9

**Published:** 2018-02-02

**Authors:** Zhengcao Cao, Yamin Wang, Liang Zhang

**Affiliations:** 1 Beijing Key Laboratory of Learning and Cognition, Department of Psychology, Capital Normal University, Beijing, 100037 China; 2 State Key Laboratory of Brain and Cognitive Science, Institute of Psychology, Chinese Academy of Sciences, Beijing, 100101 China; 3 University of Chinese Academy of Sciences, Beijing, 100049 China

Correction to: *Scientific Reports* 10.1038/s41598-017-14910-y, published online 03 November 2017

The original HTML version of this Article contained a typographical error in the publication date ‘03 November 2017’ which was incorrectly given as ‘06 November 2017’. This has now been corrected in the HTML version of the Article.

