# Author Correction: Real-time Acute Stress Facilitates Allocentric Spatial Processing in a Virtual Fire Disaster

**DOI:** 10.1038/s41598-019-40289-z

**Published:** 2019-03-12

**Authors:** Zhengcao Cao, Yamin Wang, Liang Zhang

**Affiliations:** 1 Beijing Key Laboratory of Learning and Cognition, Department of Psychology, Capital Normal University, Beijing, 100037 China; 2 State Key Laboratory of Brain and Cognitive Science, Institute of Psychology, Chinese Academy of Sciences, Beijing, 100101 China; 3 University of Chinese Academy of Sciences, Beijing, 100049 China

Correction to: *Scientific Reports* 10.1038/s41598-017-14910-y, published online 03 November 2017

The original version of this Article contained a typographical error in the Abstract.

‘In Experiment 2, 64 participants (32 males, 34 females; aged from 19 to 30) performed the SRRL task in both a low-stress VRE (a mini virtual arena) and a high-stress VRE (mini virtual arena with a fire disaster).’

now reads:

‘In Experiment 2, 66 participants (32 males, 34 females; aged from 19 to 30) performed the SRRL task in both a low-stress VRE (a mini virtual arena) and a high-stress VRE (mini virtual arena with a fire disaster).’

In addition, the original version of this Article contained a typographical error in the Author Contributions section.

‘Z.C. and Y.M. conducted the experiment and wrote the manuscript; Z.C. created all the virtual reality environments and undertook the coding of VR experiments and the data analysis. Z.C. also took the figures (Fig. 1, Fig. 2, and Fig. 3) and supplementary videos (S1, S2 and S3). Y.M. and L.Z. interpreted the results and revised the manuscript.’

now reads:

‘Z.C. and Y.W. conducted the experiment and wrote the manuscript; Z.C. created all the virtual reality environments and undertook the coding of VR experiments and the data analysis. Z.C. also took the figures (Fig. 1, Fig. 2, and Fig. 3) and supplementary videos (S1, S2 and S3). Y.W. and L.Z. interpreted the results and revised the manuscript.’

