# Supplementary material for: Real-time Acute Stress Facilitates Allocentric Spatial Processing in a Virtual Fire Disaster
Source: Sci Rep. 2017 Nov 3;7:14616. doi: 10.1038/s41598-017-14910-y (PMC5668298; doi:10.1038/s41598-017-14910-y)
Supplement: Supplementary file 1 — Supplementary Information [file 41598_2017_14910_MOESM1_ESM.pdf]

Real-time Acute Stress Facilitates Allocentric Spatial Processing in a Virtual Fire Disaster

Zhengcao Cao[1], Yamin Wang[1] & Liang Zhang[2, 3]

[1]Beijing Key Laboratory of Learning and Cognition, Department of Psychology, Capital Normal University, Beijing, 100048, China

[2]State Key Laboratory of Brain and Cognitive Science, Institute of Psychology, Chinese Academy of Sciences, Beijing, 100101, China;

[3]University of Chinese Academy of Sciences, Beijing, 100049, China

Address for correspondence:

Dr. Yamin Wang

Department of Psychology, Capital Normal University,

23A FuWai Street, Haidian District, Beijing 100048, China

E-mail: wangym@cnu.edu.cn

Supplementary information

Supplementary Video S1. A demonstration of Normal Virtual Reality Environment.

Supplementary Video S2. A demonstration of low-stress Virtual Reality Environment.

Supplementary Video S3. A demonstration of high-stress Virtual Reality Environment.
